# Supplementary figures and images for: Mitogenomes illuminate the origin and migration patterns of the indigenous people of the Canary Islands
Source: PLoS One. 2019 Mar 20;14(3):e0209125. doi: 10.1371/journal.pone.0209125 (PMC6426200; doi:10.1371/journal.pone.0209125)

A

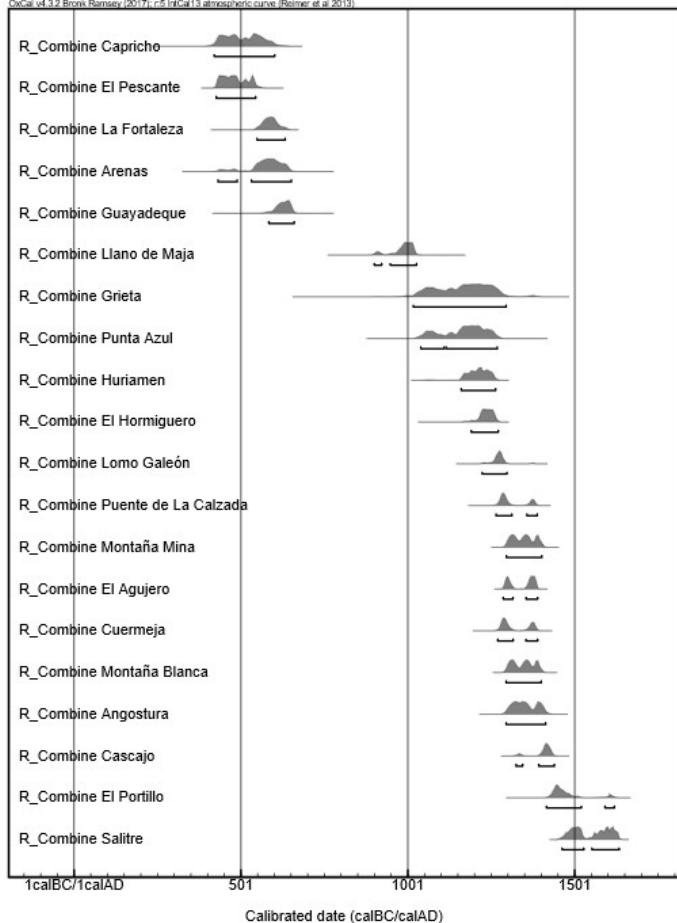

B

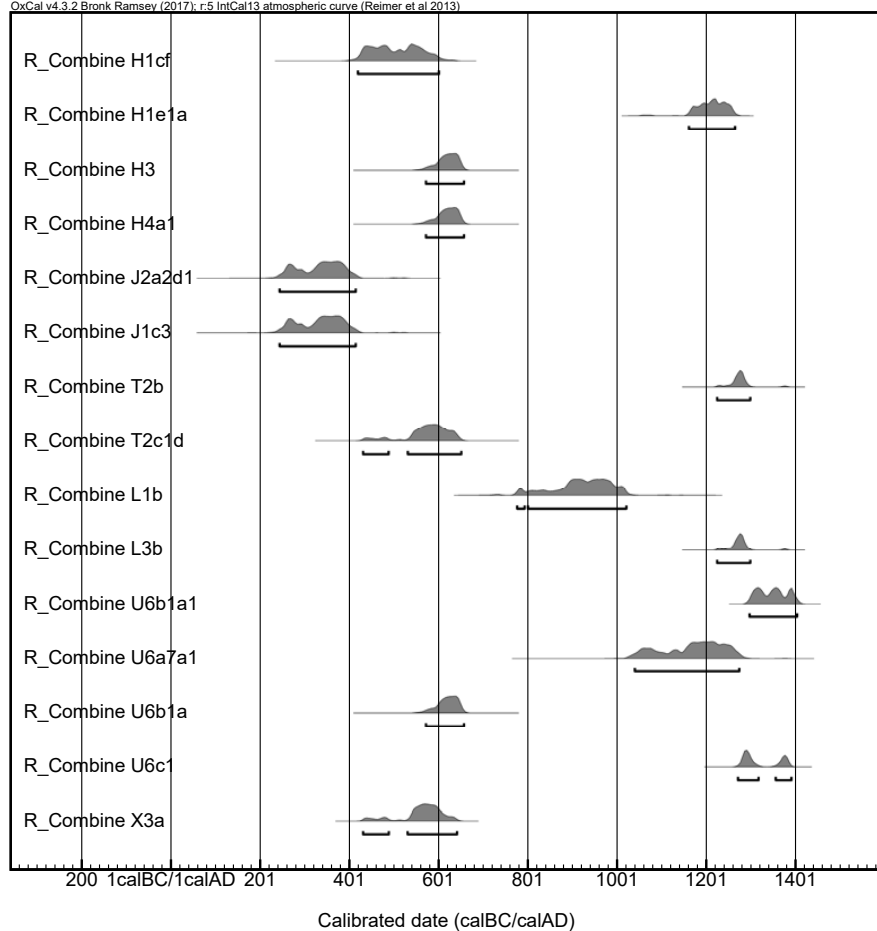

Supplement: S1 Fig — Combined calibrated radiocarbon per archaeological site (A) and per mtDNA lineage (B). (PDF) [file pone.0209125.s007.pdf]

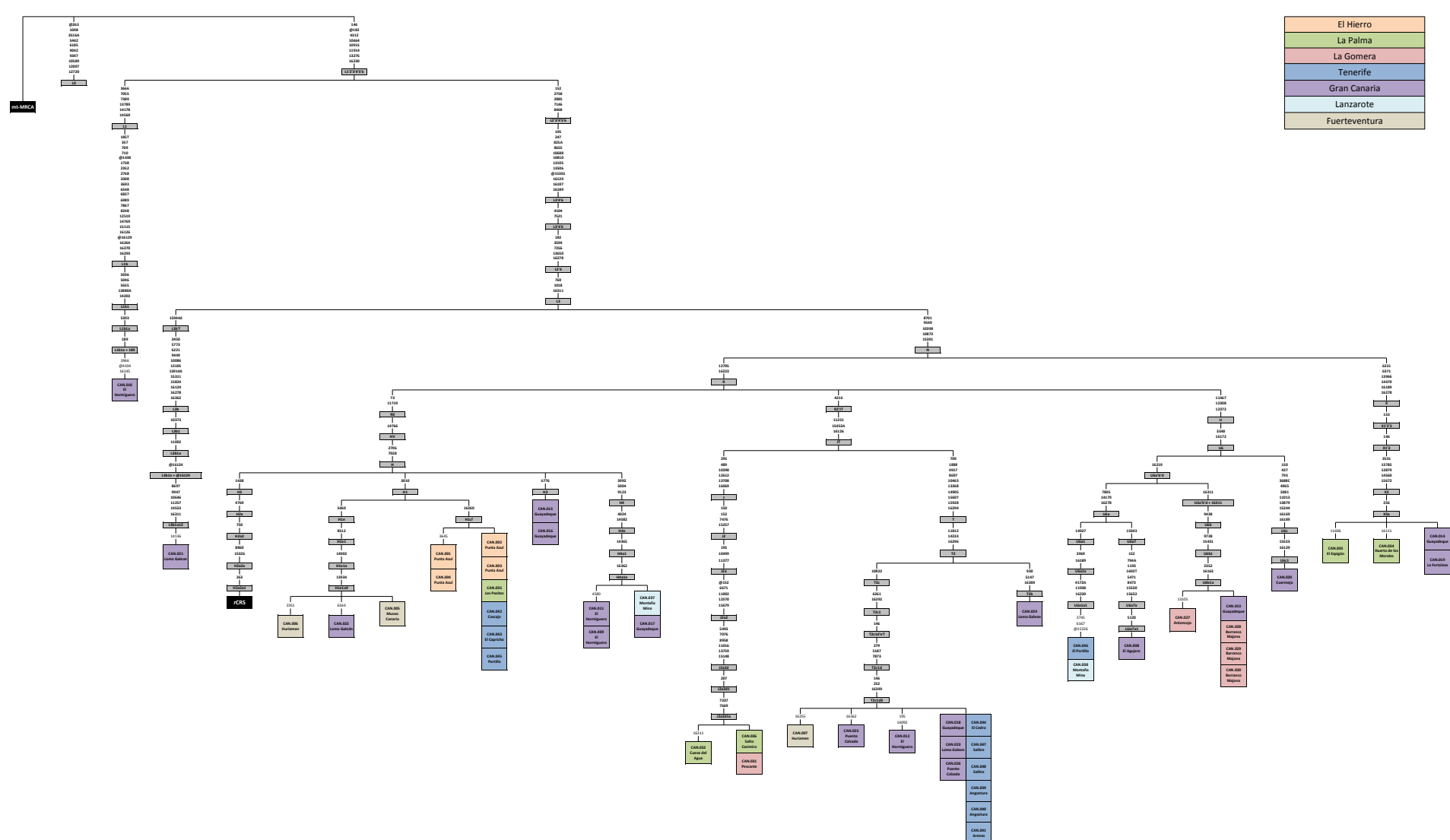

Supplement: S2 Fig — Number along links refers to nucleotide changes, whereas “@”, “d” and “i” indicates back mutations, deletions and insertions, respectively. Recurrent mutations, such as 309iC, 315iC and 16519, have not been taken into account. (PDF) [file pone.0209125.s008.pdf]

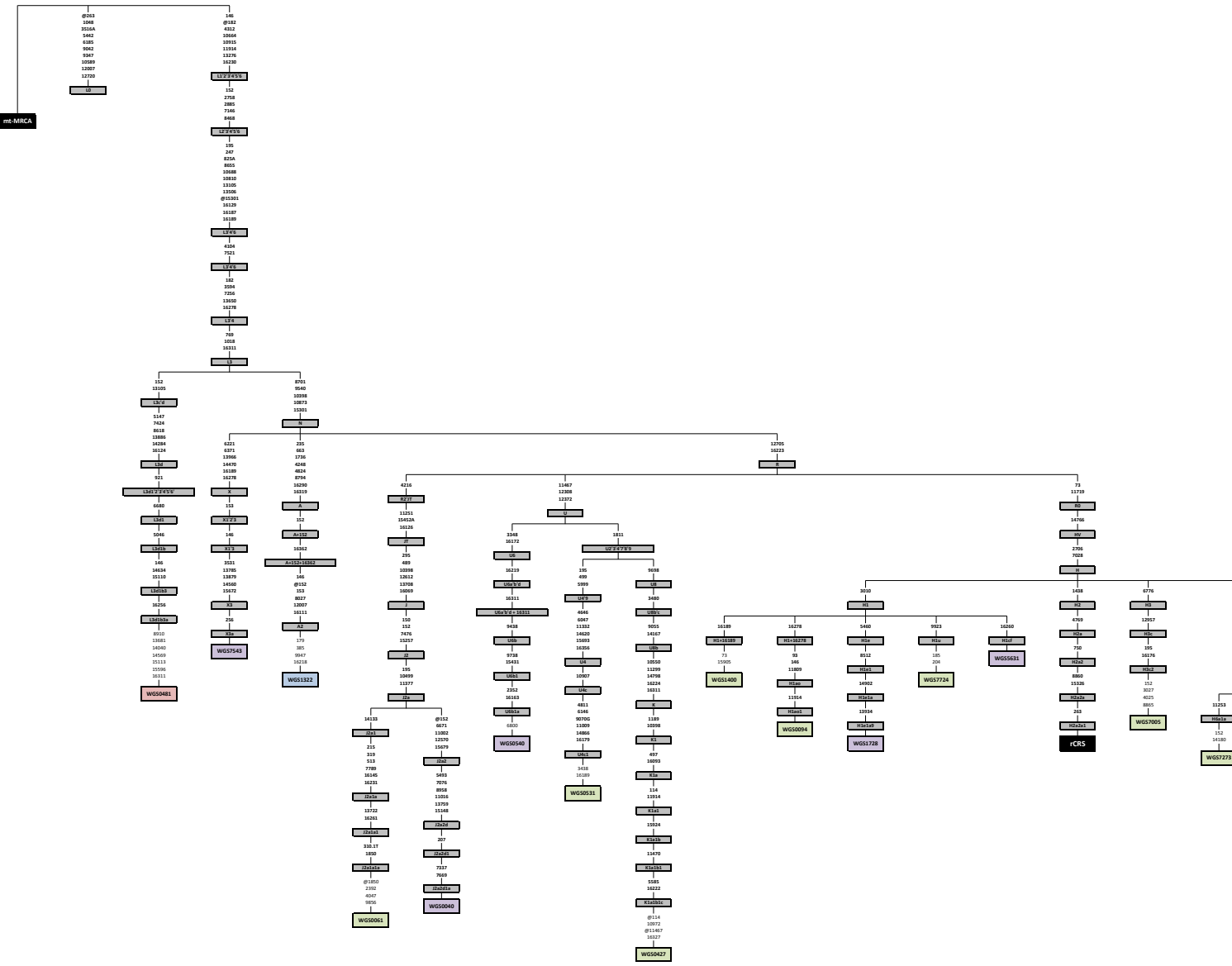

WGS1996

Supplement: S3 Fig — The most probable geographic origin of the sequences is indicated using a color code. (PDF) [file pone.0209125.s009.pdf]

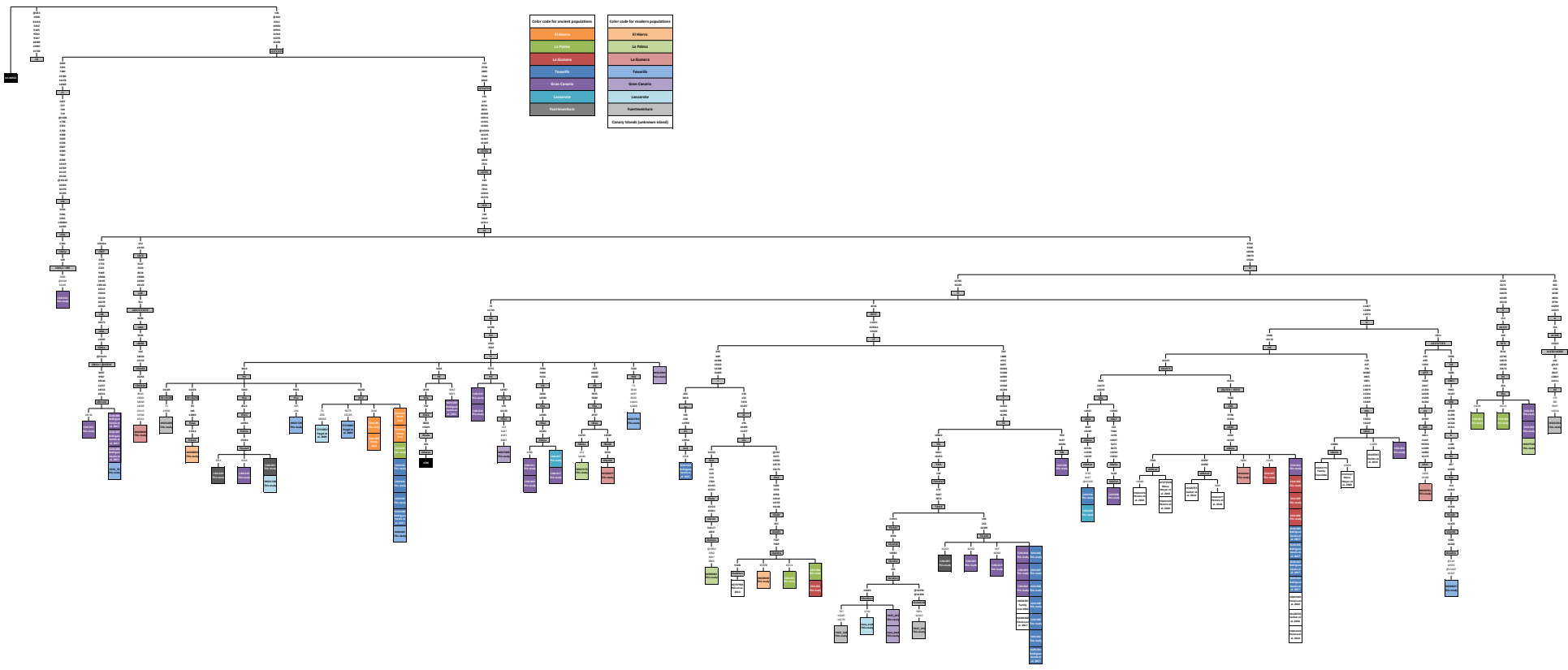

Supplement: S4 Fig — The island of origin and time period (ancient vs. modern) are indicated using a color code. (PDF) [file pone.0209125.s010.pdf]

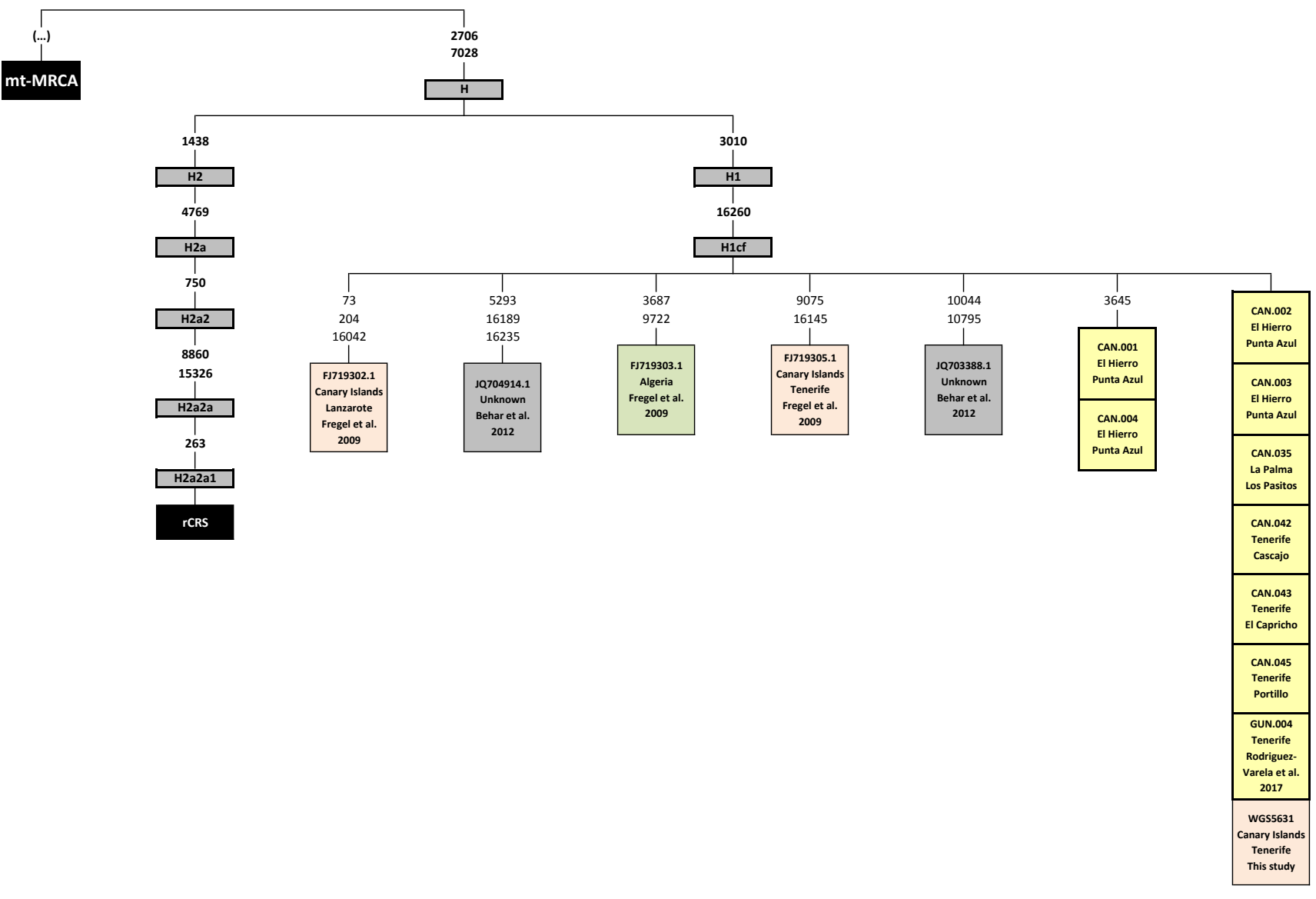

Supplement: S5 Fig — GenBank accessions and geographic origin are indicated for each complete sequence taken from the bibliography. The origin of the samples is indicated using a color code: the indigenous people of the Canary Islands (yellow); modern Canarians (orange); North Africans (green); Europeans (light blue); sub-Saharan Africans (red); the Near East (violet); the Americas (brown) and other ancient populations (blue). (PDF) [file pone.0209125.s011.pdf]

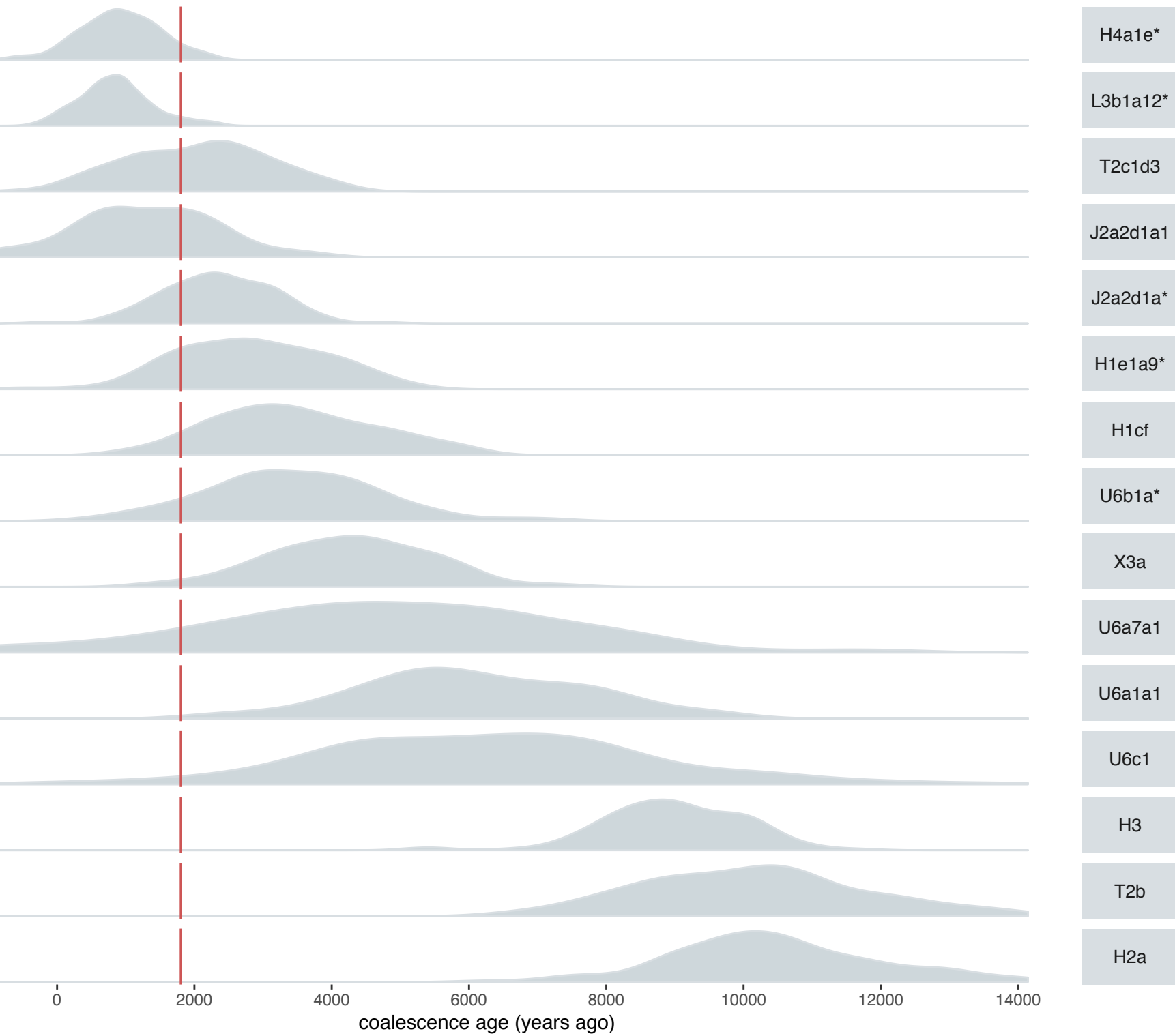

Supplement: S6 Fig — All the coalescence ages have been calculated in this study, except for H2a, H3 and T2b, whose ages have been obtained from previous results (Behar et al. 2008). The red dotted line correspond with the colonization time of the Canary Islands based on radiocarbon data. Haplogroups considered as autochthonous of the Canary Islands are indicated with an asterisk. (PDF) [file pone.0209125.s012.pdf]

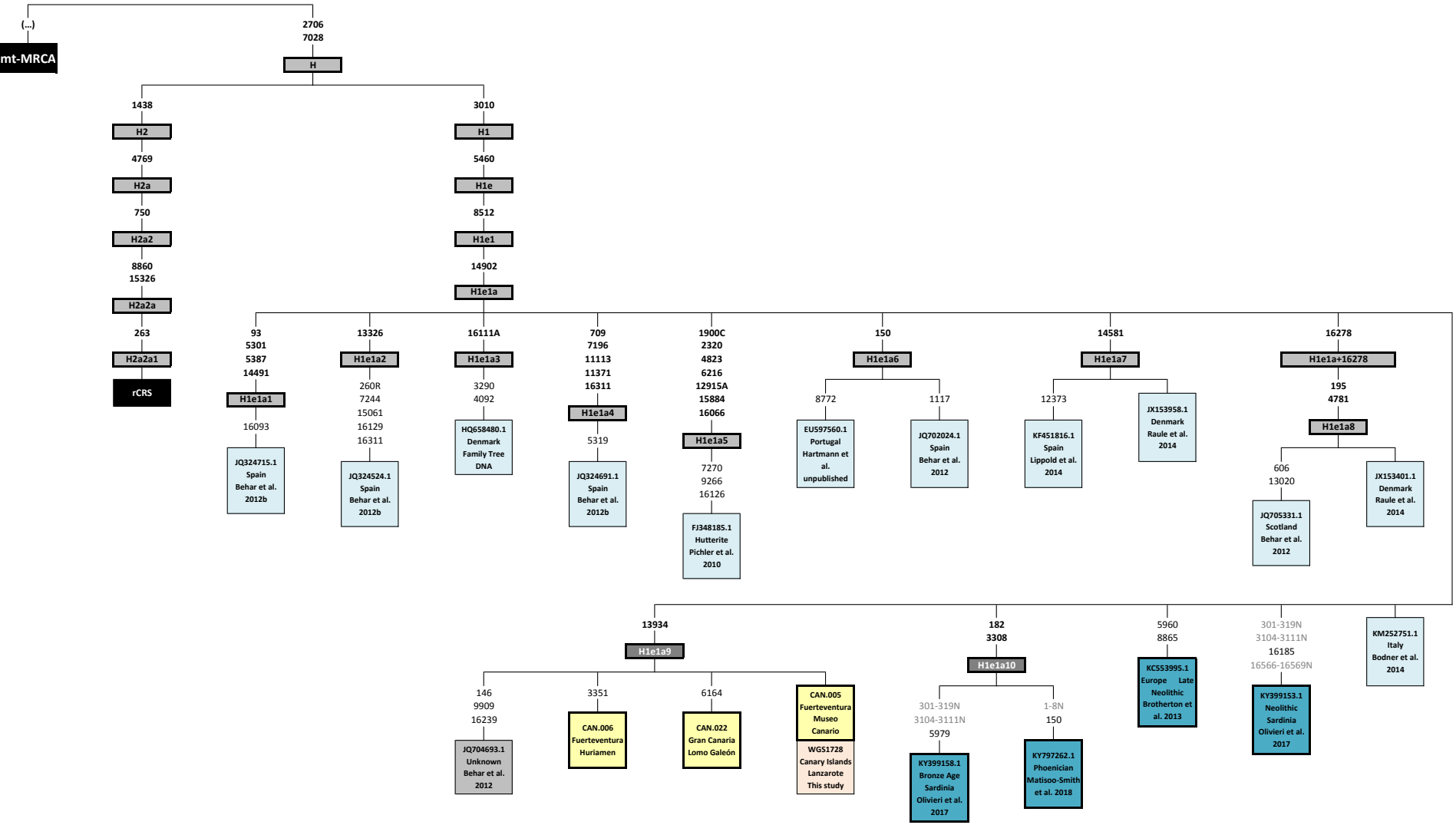

Supplement: S7 Fig — GenBank accessions and geographic origin are indicated for each complete sequence taken from the bibliography. Sub-haplogroups in dark grey and white fonts indicate newly defined branches. Color codes are as in S5 Fig. (PDF) [file pone.0209125.s013.pdf]

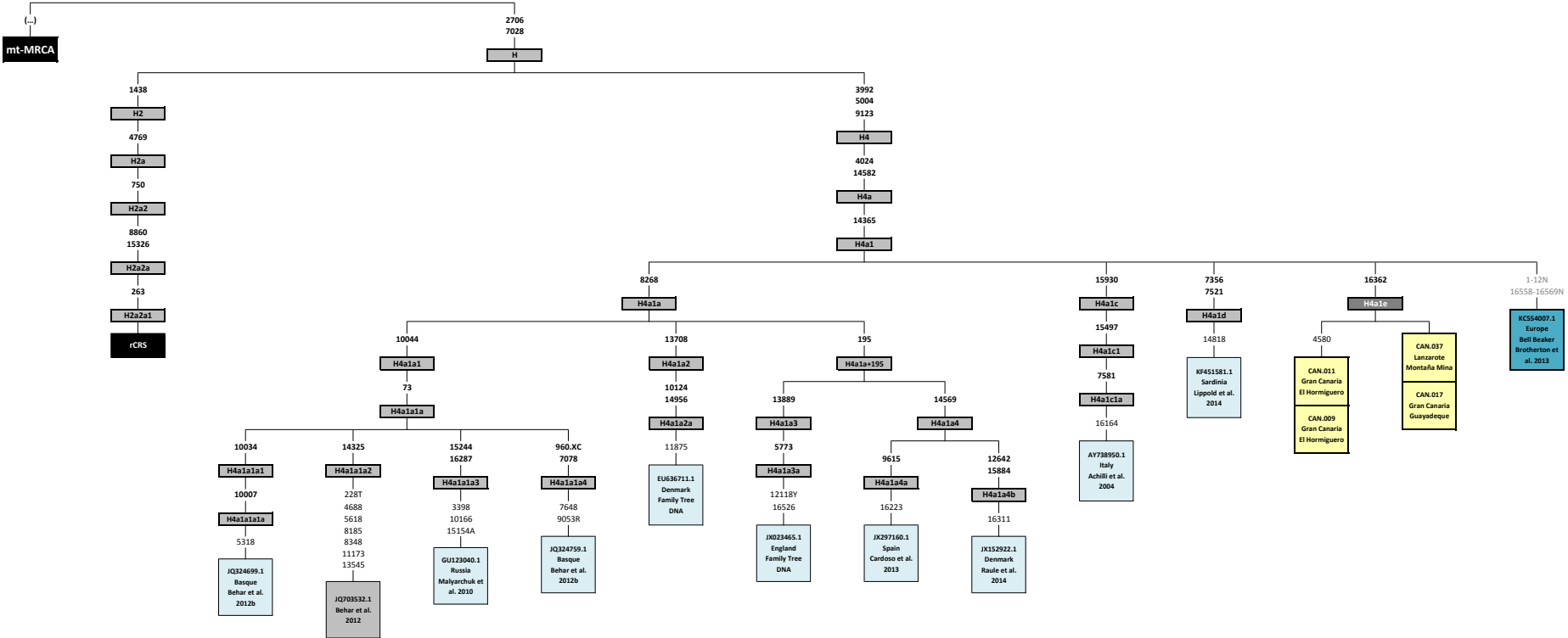

Supplement: S8 Fig — GenBank accessions and geographic origin are indicated for each complete sequence taken from the bibliography. Sub-haplogroups in dark grey and white fonts indicate newly defined branches. Color codes are as in S5 Fig. (PDF) [file pone.0209125.s014.pdf]

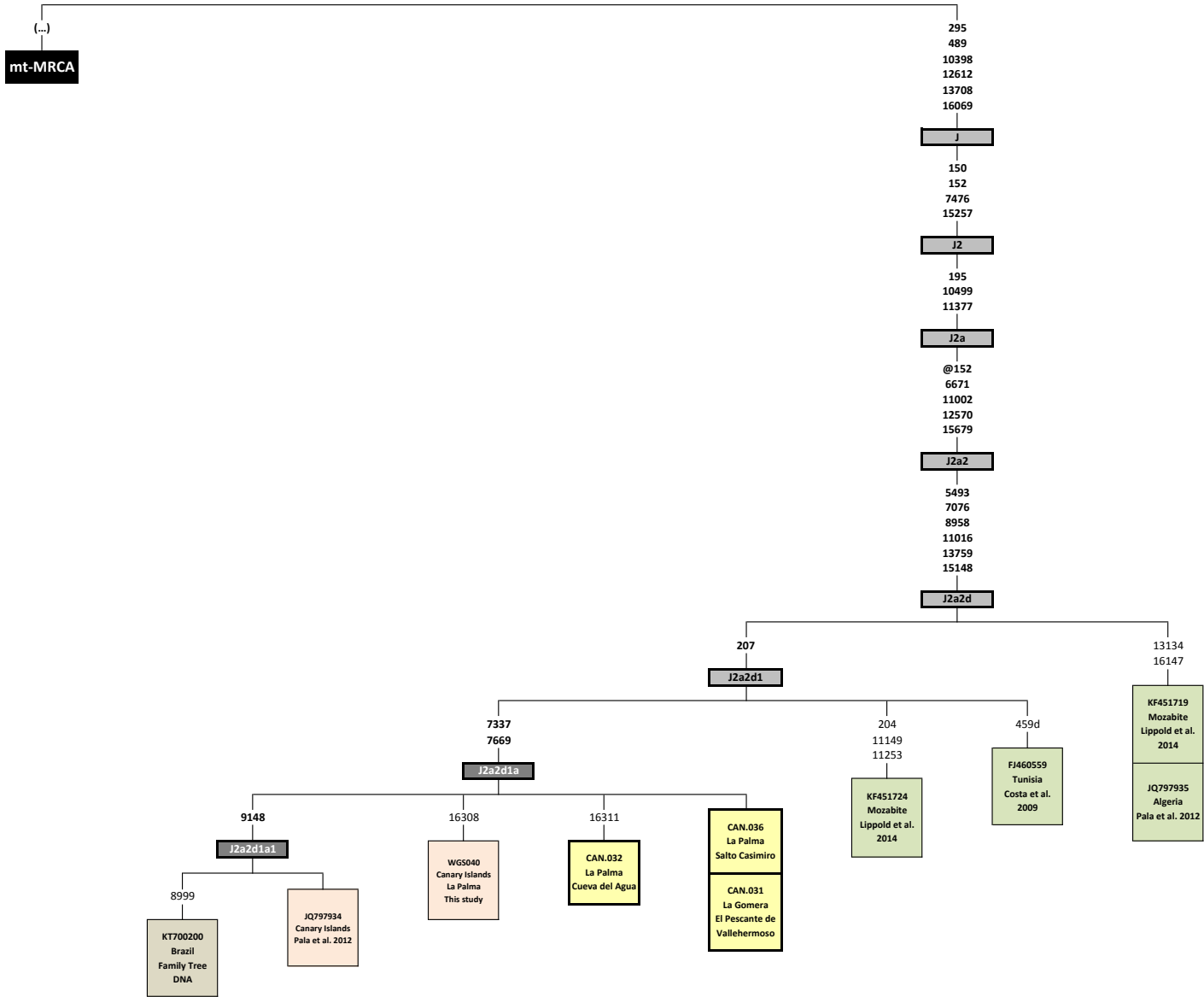

Supplement: S10 Fig — GenBank accessions and geographic origin are indicated for each complete sequence taken from the bibliography. Sub-haplogroups in dark grey and white fonts indicate newly defined branches. Color codes are as in S5 Fig. (PDF) [file pone.0209125.s016.pdf]

mt-MRCA

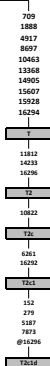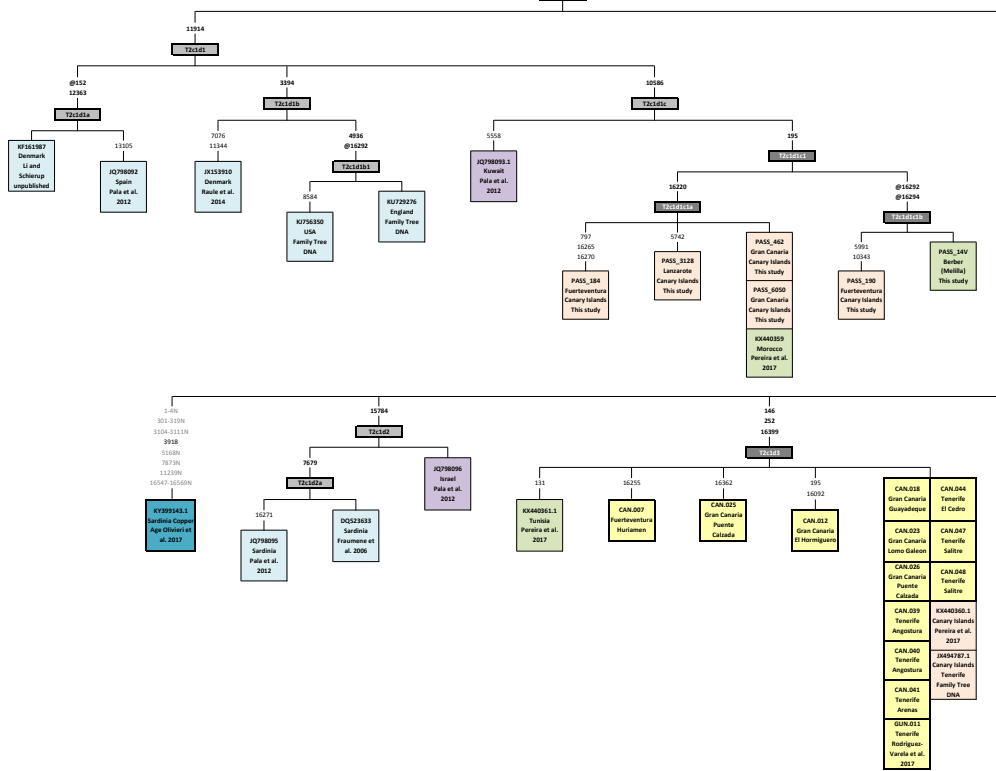

Supplement: S11 Fig — GenBank accessions and geographic origin are indicated for each complete sequence taken from the bibliography. Sub-haplogroups in dark grey and white fonts indicate newly defined branches. Color codes are as in S5 Fig. (PDF) [file pone.0209125.s017.pdf]

(...)  
mt-MRCA

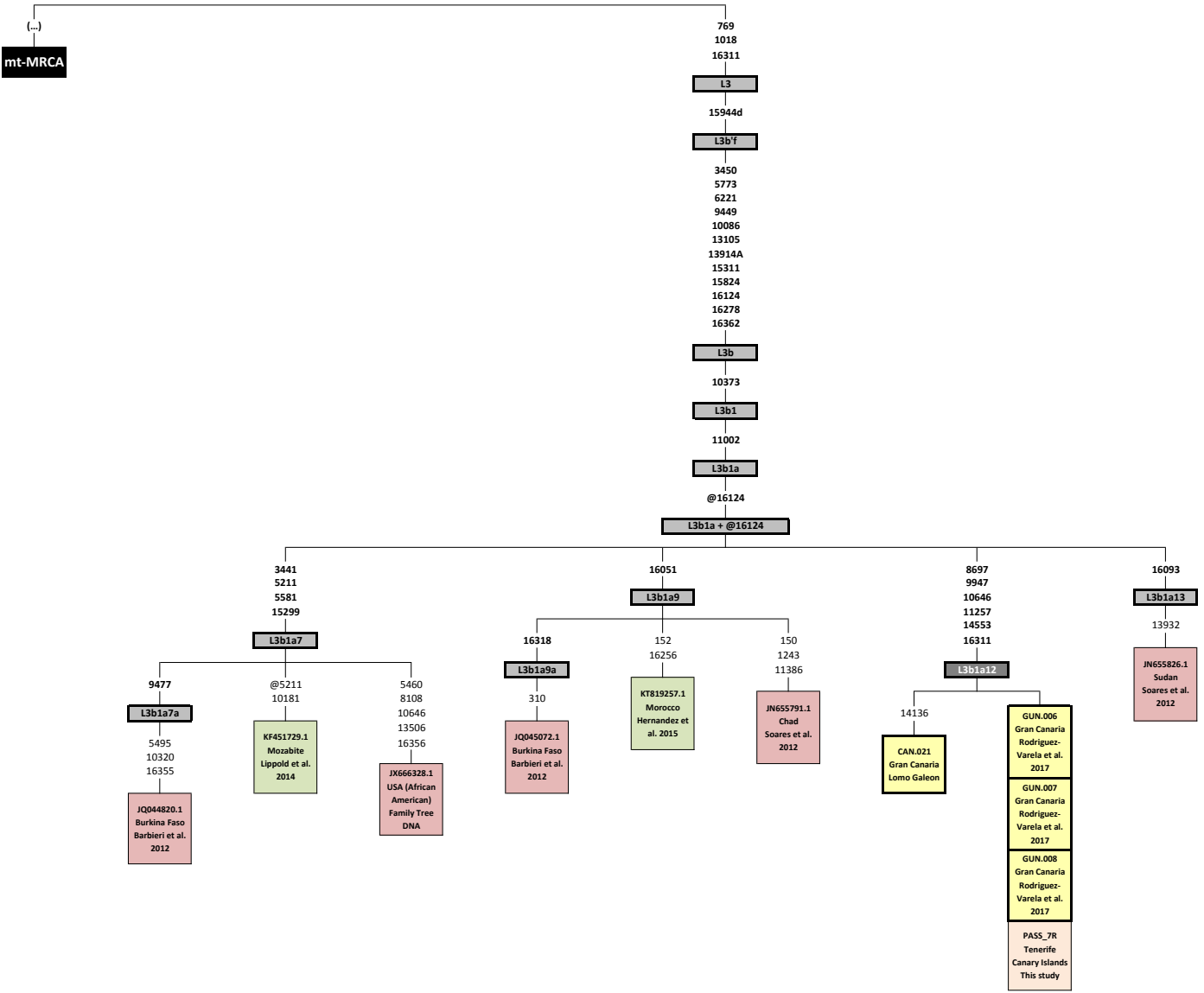

Supplement: S12 Fig — GenBank accessions and geographic origin are indicated for each complete sequence taken from the bibliography. Sub-haplogroups in dark grey and white fonts indicate newly defined branches. Color codes are as in S5 Fig. (PDF) [file pone.0209125.s018.pdf]

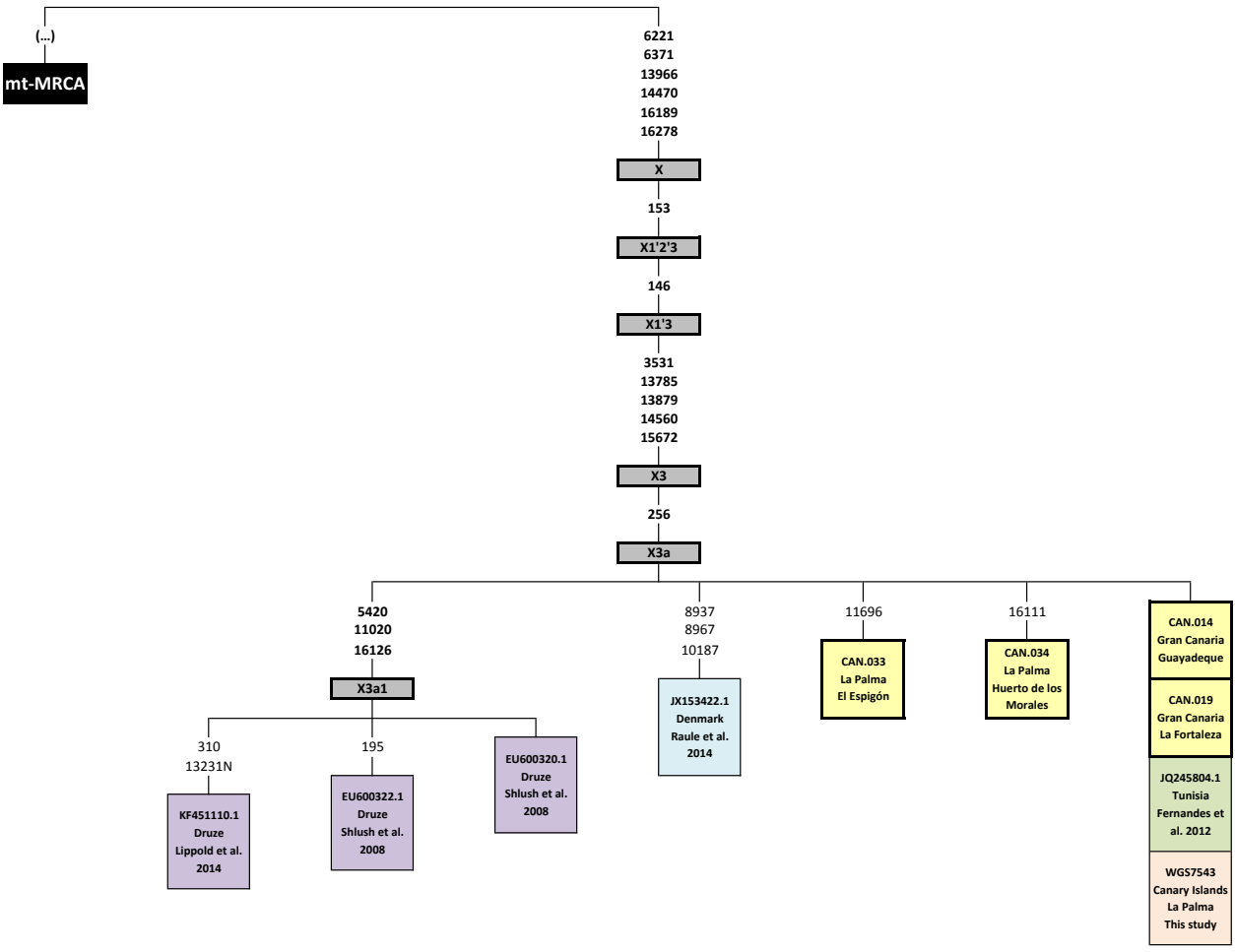

Supplement: S13 Fig — GenBank accessions and geographic origin are indicated for each complete sequence taken from the bibliography. Color codes are as in Figure S5. (PDF) [file pone.0209125.s019.pdf]

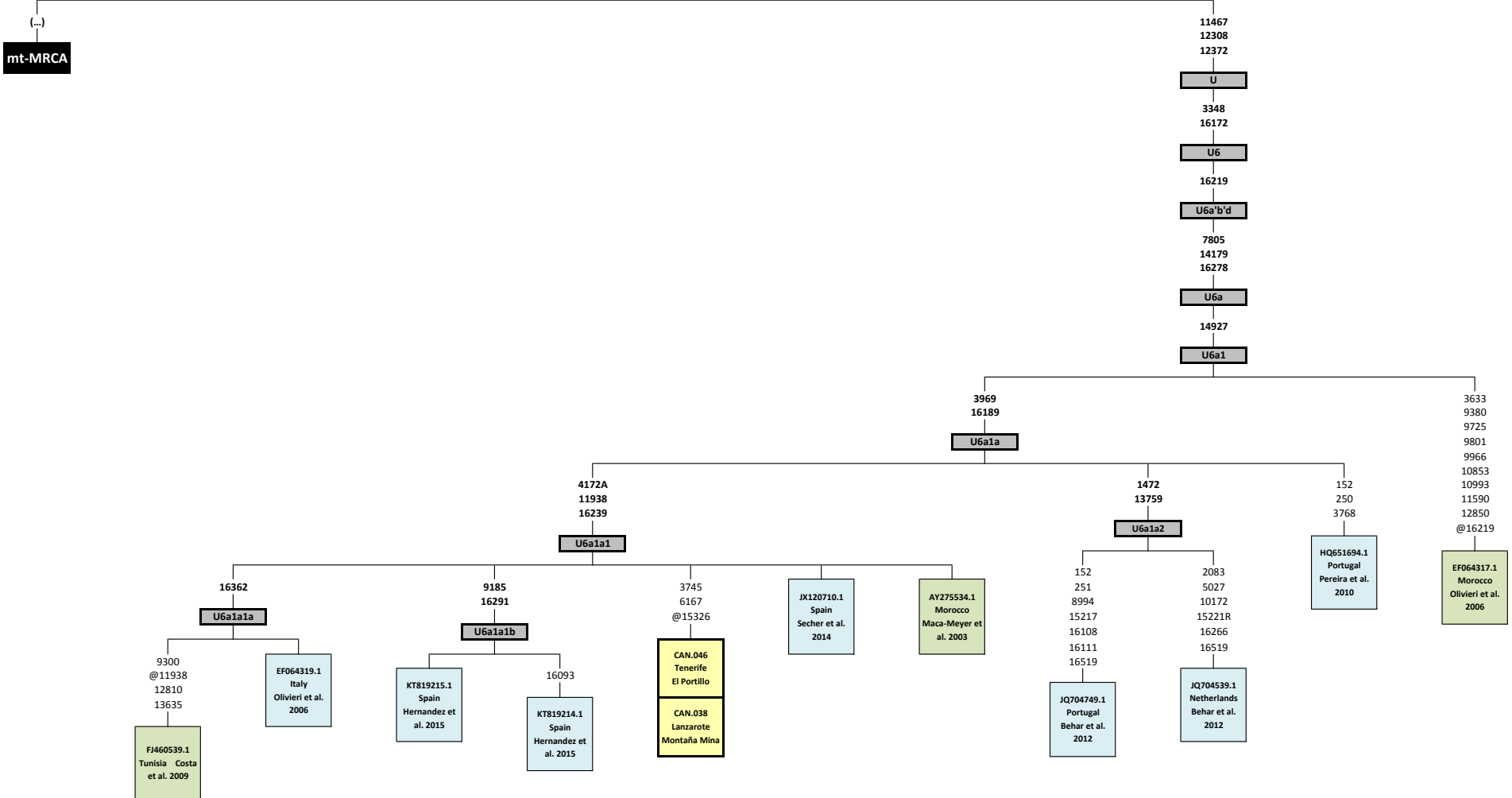

Supplement: S14 Fig — GenBank accessions and geographic origin are indicated for each complete sequence taken from the bibliography. Color codes are as in Figure S5. (PDF) [file pone.0209125.s020.pdf]

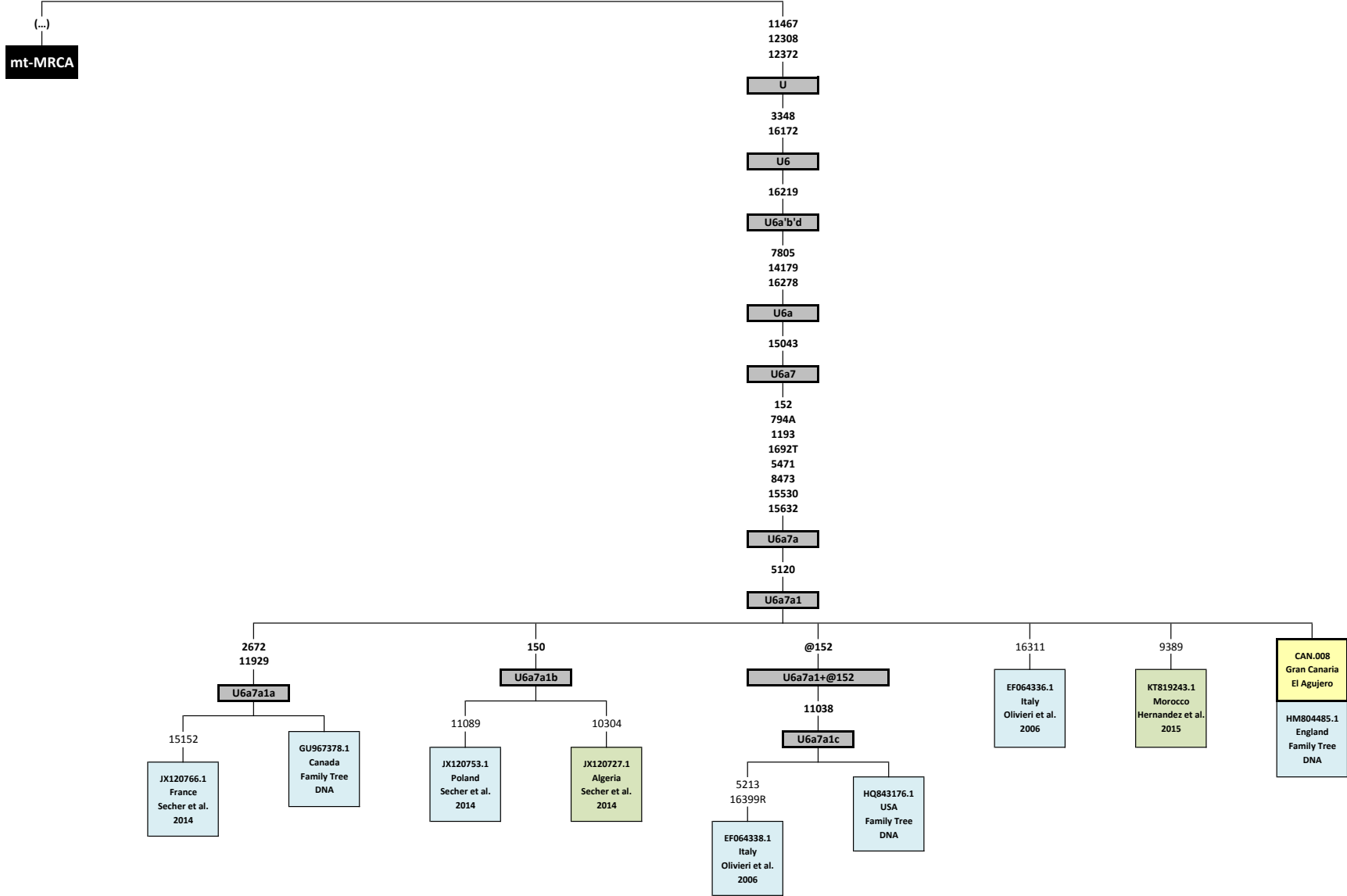

Supplement: S15 Fig — GenBank accessions and geographic origin are indicated for each complete sequence taken from the bibliography. Color codes are as in Figure S5. (PDF) [file pone.0209125.s021.pdf]

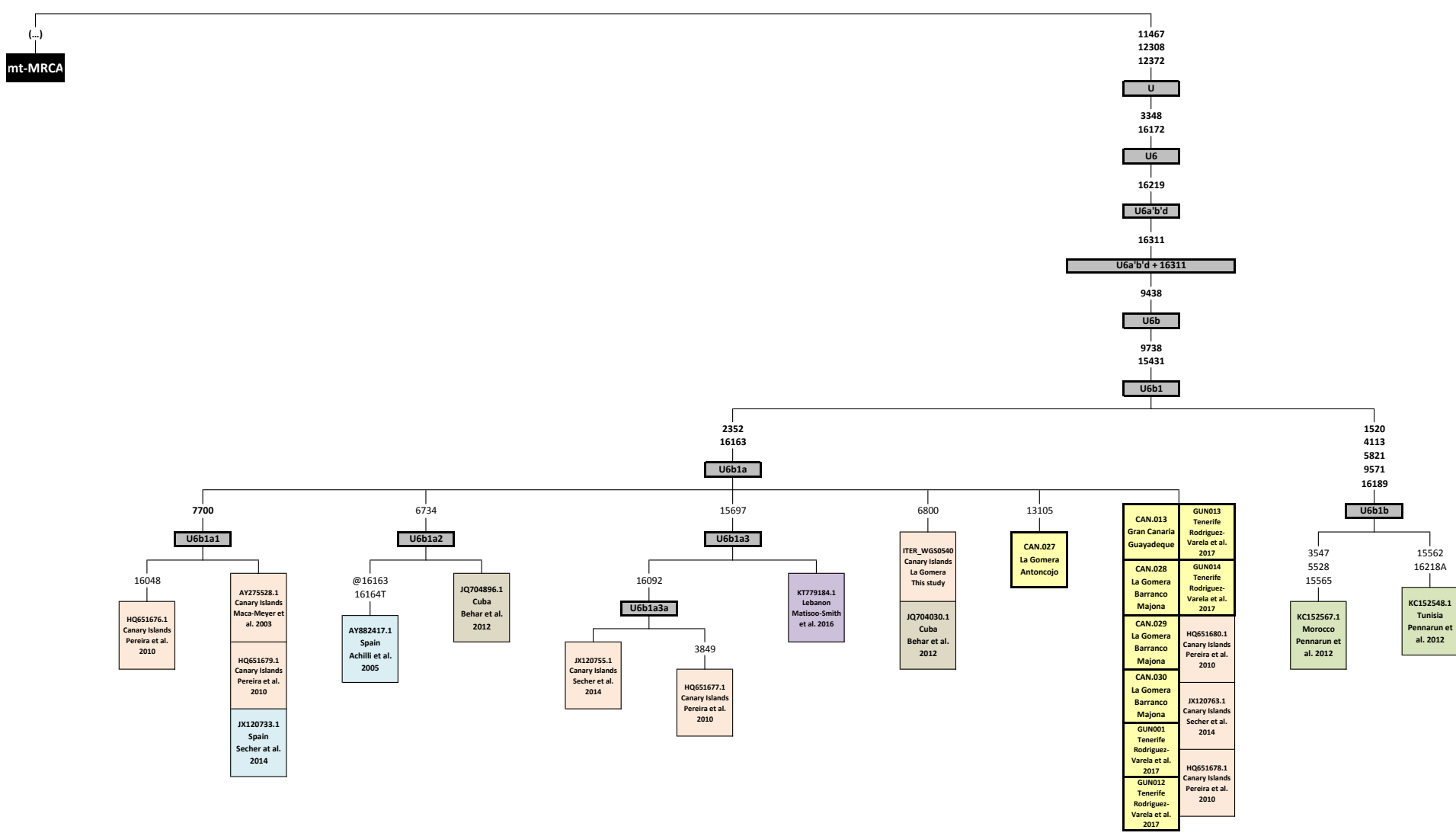

Supplement: S16 Fig — GenBank accessions and geographic origin are indicated for each complete sequence taken from the bibliography. Color codes are as in Figure S5. (PDF) [file pone.0209125.s022.pdf]

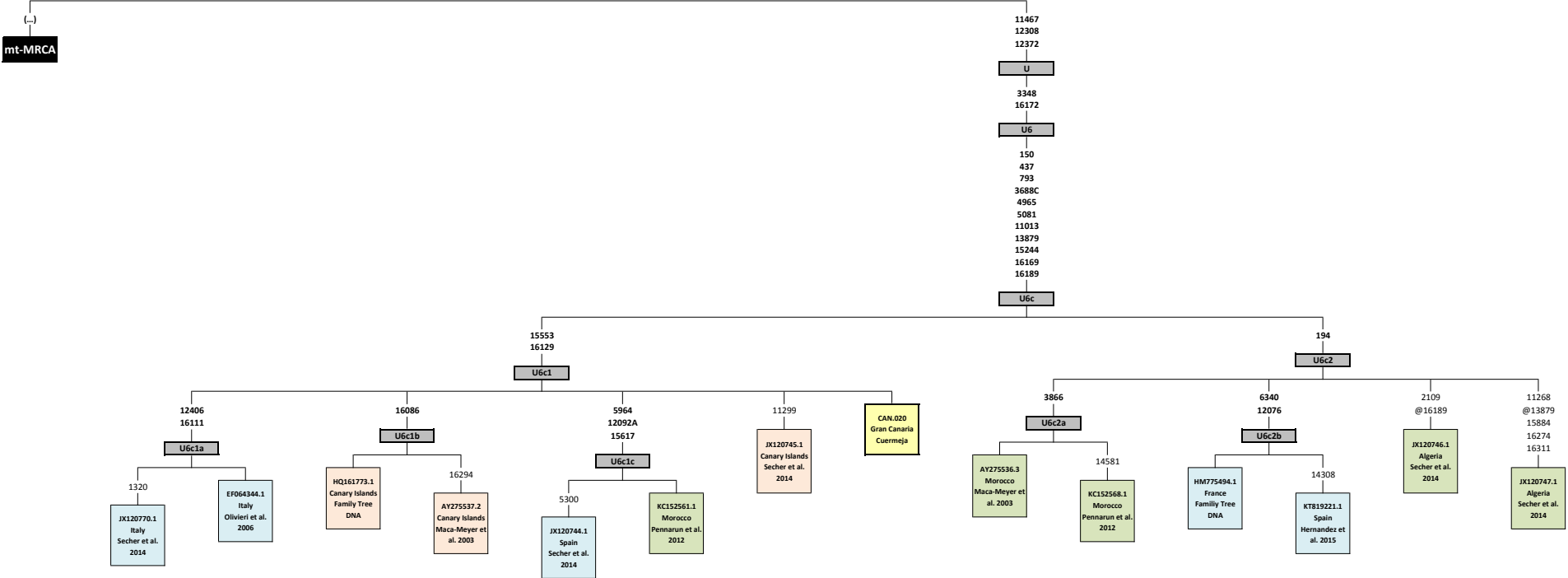

Supplement: S17 Fig — GenBank accessions and geographic origin are indicated for each complete sequence taken from the bibliography. Color codes are as in Figure S5. (PDF) [file pone.0209125.s023.pdf]
