# Supplementary material for: Mitogenomes illuminate the origin and migration patterns of the indigenous people of the Canary Islands
Source: PLoS One. 2019 Mar 20;14(3):e0209125. doi: 10.1371/journal.pone.0209125 (PMC6426200; doi:10.1371/journal.pone.0209125)
Supplement: S9 Fig — GenBank accessions and geographic origin are indicated for each complete sequence taken from the bibliography. Color codes are as in Figure S5. (PDF) [file pone.0209125.s015.pdf]

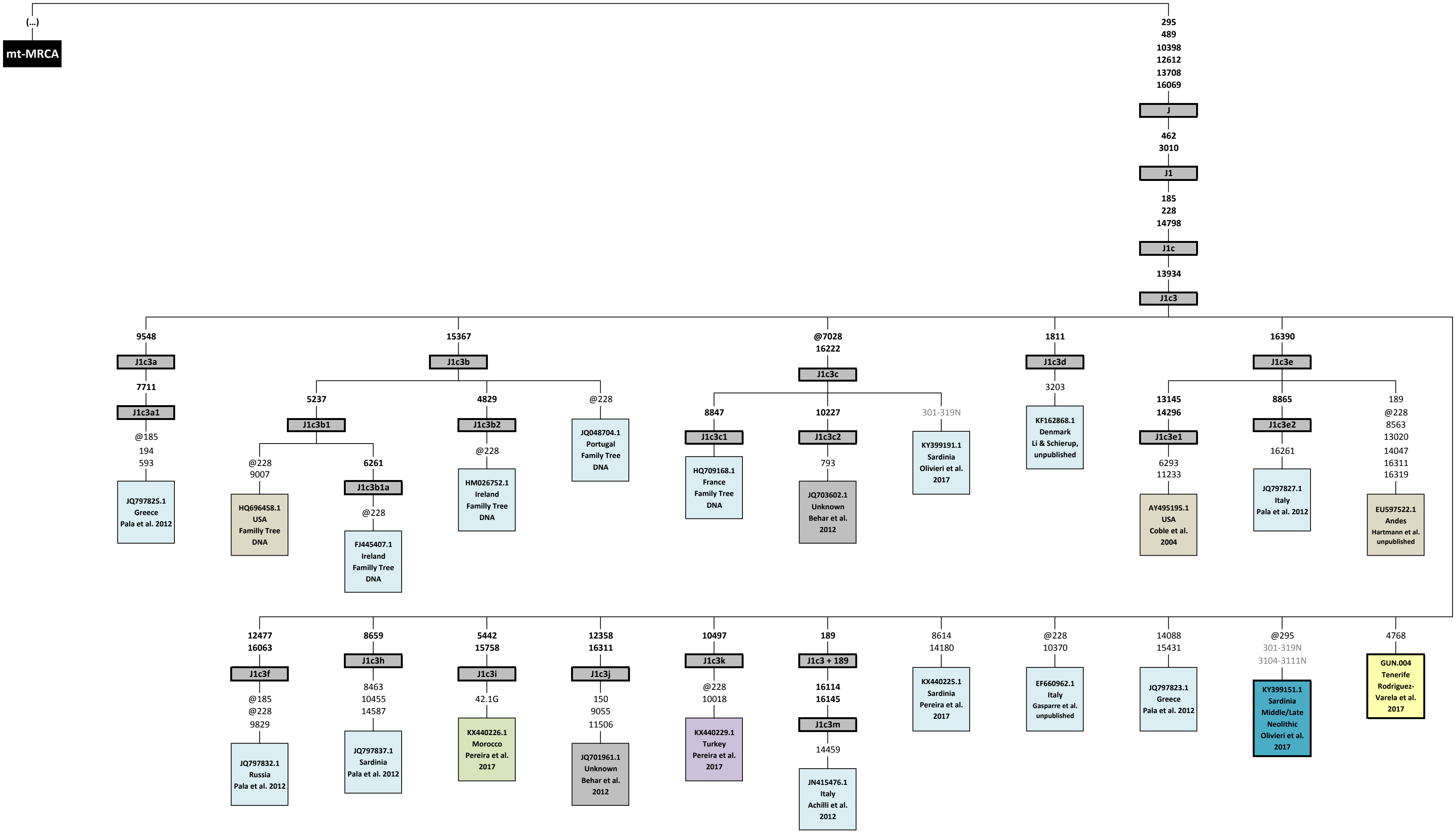
(...)
mt-MRCA
295
489
10398
12612
13708
16069
J
462
3010
J1
185
228
14798
J1c
13934
J1c3
9548
J1c3a
7711
J1c3a1
@185
194
593
JQ797825.1
Greece
Pala et al. 2012
15367
J1c3b
5237
J1c3b1
@228
9007
HQ696458.1
USA
Familly Tree
DNA
6261
J1c3b1a
@228
FJ445407.1
Ireland
Familly Tree
DNA
4829
J1c3b2
@228
HM026752.1
Ireland
Familly Tree
DNA
@228
JQ048704.1
Portugal
Family Tree
DNA
@7028
16222
J1c3c
8847
J1c3c1
HQ709168.1
France
Family Tree
DNA
10227
J1c3c2
793
JQ703602.1
Unknown
Behar et al.
2012
301-319N
KY399191.1
Sardinia
Olivieri et al.
2017
1811
J1c3d
3203
KF162868.1
Denmark
Li & Schierup,
unpublished
16390
J1c3e
13145
14296
J1c3e1
6293
11233
AY495195.1
USA
Coble et al.
2004
8865
J1c3e2
16261
JQ797827.1
Italy
Pala et al. 2012
189
@228
8563
13020
14047
16311
16319
EU597522.1
Andes
Hartmann et al.
unpublished
12477
16063
J1c3f
@185
@228
9829
JQ797832.1
Russia
Pala et al. 2012
8659
J1c3h
8463
10455
14587
JQ797837.1
Sardinia
Pala et al. 2012
5442
15758
J1c3i
42.1G
KX440226.1
Morocco
Pereira et al.
2017
12358
16311
J1c3j
150
9055
11506
JQ701961.1
Unknown
Behar et al.
2012
10497
J1c3k
@228
10018
KX440229.1
Turkey
Pereira et al.
2017
189
J1c3 + 189
16114
16145
J1c3m
14459
JN415476.1
Italy
Achilli et al.
2012
8614
14180
KX440225.1
Sardinia
Pereira et al.
2017
@228
10370
EF660962.1
Italy
Gasparre et al.
unpublished
14088
15431
JQ797823.1
Greece
Pala et al. 2012
@295
301-319N
3104-3111N
KY399151.1
Sardinia
Middle/Late
Neolithic
Olivieri et al.
2017
4768
GUN.004
Tenerife
Rodriguez-
Varela et al.
2017
