# Supplementary material for: Mitogenomes illuminate the origin and migration patterns of the indigenous people of the Canary Islands
Source: PLoS One. 2019 Mar 20;14(3):e0209125. doi: 10.1371/journal.pone.0209125 (PMC6426200; doi:10.1371/journal.pone.0209125)
Supplement: S1 File — (PDF) [file pone.0209125.s024.pdf]

haplotype ID	mitogenome ID	island	site	specimen information	repository institution
FUI01	CAN.006	Fuerteventura	Huriamen	HUR-01	Cabildo Insular de Fuerteventura
FUI02	CAN.005	Fuerteventura	Unknown	143.29	Museo Canario
FUI03	-	Fuerteventura	Unknown	CVAVARI	Cabildo Insular de Fuerteventura
FUI04	-	Fuerteventura	Montaña Tirba	TIRBA 4	Cabildo Insular de Fuerteventura
FUI05	-	Fuerteventura	Unknown	SREF 3/ CO206	Cabildo Insular de Fuerteventura
FUI06	-	Fuerteventura	Unknown	SREF2 / CO206	Cabildo Insular de Fuerteventura
FUI07	-	Fuerteventura	Montaña Tirba	TIRBA 3	Cabildo Insular de Fuerteventura
FUI08	-	Fuerteventura	Unknown	CAJA CO206 1	Cabildo Insular de Fuerteventura
FUI09	-	Fuerteventura	Unknown	Ref. 18	Cabildo Insular de Fuerteventura
FUI10	-	Fuerteventura	Unknown	FR1	Cabildo Insular de Fuerteventura
FUI11	CAN.007	Fuerteventura	Huriamen	HUR-02	Cabildo Insular de Fuerteventura
FUI12	-	Fuerteventura	Unknown	SREF CO206	Cabildo Insular de Fuerteventura
FUI13	-	Fuerteventura	Unknown	CAJA CO206 2	Cabildo Insular de Fuerteventura
GCI02	-	Gran Canaria	Crucecita	CRU04 E13	Museo Canario
GCI03	-	Gran Canaria	Crucecita	E6	Museo Canario
GCI04	-	Gran Canaria	Guayadeque	0146/61	Museo Canario
GCI05	-	Gran Canaria	Guayadeque	0162/3	Museo Canario
GCI06	-	Gran Canaria	Guayadeque	0192/40	Museo Canario
GCI07	-	Gran Canaria	Guayadeque	0192/25	Museo Canario
GCI08	-	Gran Canaria	Guayadeque	OHHCII-5	Museo Canario
GCI09	-	Gran Canaria	Guayadeque	0146/32B	Museo Canario
GCI10	-	Gran Canaria	Guayadeque	0158/SR13	Museo Canario
GCI11	-	Gran Canaria	Guayadeque	0159/114	Museo Canario
GCI12	-	Gran Canaria	Guayadeque	0159/74	Museo Canario
GCI13	-	Gran Canaria	Guayadeque	0146/51	Museo Canario
GCI14	-	Gran Canaria	Guayadeque	0206/SP10	Museo Canario
GCI15	-	Gran Canaria	Guayadeque	OHHCII-6	Museo Canario
GCI16	-	Gran Canaria	Guayadeque	suelto 3	Museo Canario
GCI17	-	Gran Canaria	Guayadeque	FMC_1	Museo Canario
GCI18	-	Gran Canaria	Guayadeque	868 completo	Museo Canario
GCI19	-	Gran Canaria	Guayadeque	suelto 1	Museo Canario
GCI20	-	Gran Canaria	Guayadeque	FMC_3	Museo Canario
GCI21	-	Gran Canaria	Guayadeque	0162/297	Museo Canario
GCI22	-	Gran Canaria	Guayadeque	0146/26	Museo Canario
GCI23	-	Gran Canaria	Guayadeque	0206/111	Museo Canario
GCI24	-	Gran Canaria	Guayadeque	0146/29	Museo Canario
GCI25	-	Gran Canaria	Guayadeque	0206/SR22	Museo Canario
GCI26	-	Gran Canaria	Guayadeque	0206/SR20	Museo Canario
GCI27	-	Gran Canaria	Guayadeque	0162/SR4	Museo Canario
GCI28	-	Gran Canaria	Guayadeque	0146/43	Museo Canario
GCI29	CAN.015	Gran Canaria	Guayadeque	0158/SR11	Museo Canario
GCI30	CAN.016	Gran Canaria	Guayadeque	0206/SR21B	Museo Canario
GCI31	-	Gran Canaria	Lomo Caserones	Individuo 4	Museo Canario
GCI32	CAN.022	Gran Canaria	Lomo Galeón	Ref. 24 Caja 269	Museo Canario
GCI33	-	Gran Canaria	Guayadeque	0192/4	Museo Canario
GCI34	CAN.009	Gran Canaria	El Hormiguero	Individuo 10	Museo Canario
GCI35	CAN.011	Gran Canaria	El Hormiguero	Individuo 6	Museo Canario
GCI36	CAN.017	Gran Canaria	Guayadeque	0192/39	Museo Canario
GCI37	-	Gran Canaria	Guayadeque	0192/870	Museo Canario
GCI38	-	Gran Canaria	Restinga	RESTINGA	Museo Canario
GCI39	-	Gran Canaria	Guayadeque	0162/100	Museo Canario
GCI40	-	Gran Canaria	Guayadeque	suelto 4	Museo Canario
GCI41	CAN.010	Gran Canaria	El Hormiguero	Individuo 1	Museo Canario
GCI42	-	Gran Canaria	Guayadeque	0146/SR14	Museo Canario
GCI46	-	Gran Canaria	Guayadeque	OHHCI-2	Museo Canario
GCI47	CAN.021	Gran Canaria	Lomo Galeón	Ref.99, Caja 271bis	Museo Canario
GCI48	-	Gran Canaria	Maspalomas	B66	Museo Canario
GCI49	-	Gran Canaria	Guayadeque	0162/105	Museo Canario
GCI50	CAN.024	Gran Canaria	Lomo Galeón	Ref. 45, Caja 270	Museo Canario
GCI51	-	Gran Canaria	Maspalomas	B15-112 Individuo 1	Museo Canario
GCI52	-	Gran Canaria	El Agujero	881 completo	Museo Canario
GCI53	CAN.012	Gran Canaria	El Hormiguero	Individuo 2	Museo Canario
GCI54	-	Gran Canaria	Guayadeque	0206/SP3	Museo Canario
GCI55	-	Gran Canaria	Guayadeque	0192/SR	Museo Canario
GCI56	-	Gran Canaria	Guayadeque	1062/SR5	Museo Canario
GCI57	CAN.023	Gran Canaria	Lomo Galeón	Ref.79, Caja 71bis	Museo Canario
GCI58	-	Gran Canaria	Guayadeque	0159/SR8	Museo Canario

haplotype ID	mitogenome ID	island	site	specimen information	repository institution
GCI59	-	Gran Canaria	Guayadeque	0159/SR6	Museo Canario
GCI60	-	Gran Canaria	Guayadeque	suelto 2	Museo Canario
GCI61	-	Gran Canaria	Guayadeque	0192/24	Museo Canario
GCI62	CAN.018	Gran Canaria	Guayadeque	0146/32	Museo Canario
GCI63	-	Gran Canaria	Maspalomas	B121	Museo Canario
GCI64	-	Gran Canaria	Maspalomas	B131	Museo Canario
GCI65	CAN.026	Gran Canaria	Puente de la Calzada	Maxilar nº391	Museo Canario
GCI66	CAN.025	Gran Canaria	Puente de la Calzada	UE1 (1I , Ie)	Museo Canario
GCI67	-	Gran Canaria	Guayadeque	0162/SR2	Museo Canario
GCI68	-	Gran Canaria	Guayadeque	0159/80	Museo Canario
GCI69	-	Gran Canaria	Guayadeque	0146/37	Museo Canario
GCI70	CAN.008	Gran Canaria	El Agujero	41.566	Museo Canario
GCI71	-	Gran Canaria	Guayadeque	FMC_2	Museo Canario
GCI72	-	Gran Canaria	Guayadeque	0206/34	Museo Canario
GCI73	-	Gran Canaria	Guayadeque	0162/SR3	Museo Canario
GCI74	-	Gran Canaria	Guayadeque	0206/SR21	Museo Canario
GCI75	-	Gran Canaria	Guayadeque	0192/7	Museo Canario
GCI76	-	Gran Canaria	Guayadeque	0192/45	Museo Canario
GCI77	-	Gran Canaria	Guayadeque	0159/SR7	Museo Canario
GCI78	-	Gran Canaria	Guayadeque	FMC_4	Museo Canario
GCI79	CAN.013	Gran Canaria	Guayadeque	0146/45	Museo Canario
GCI80	CAN.020	Gran Canaria	Cuermeja	Caja 0357	Museo Canario
GCI81	-	Gran Canaria	Guayadeque	882 completo	Museo Canario
GCI82	-	Gran Canaria	Guayadeque	OHHC II-4	Museo Canario
GCI83	-	Gran Canaria	Guayadeque	0206/SR19	Museo Canario
GCI84	-	Gran Canaria	Guayadeque	0162/12	Museo Canario
GCI85	CAN.014	Gran Canaria	Guayadeque	0146/72	Museo Canario
GCI86	CAN.019	Gran Canaria	La Fortaleza	977	Museo Canario
GCI87	-	Gran Canaria	Crucecita	CRU04 E4	Museo Arqueológico de La Gomera
GOI09	CAN.031	La Gomera	Pescante	CHI-12.7	Museo Arqueológico de La Gomera
GOI50	CAN.027	La Gomera	Antoncojo	cueva	Museo Arqueológico de La Gomera
GOI51	CAN.028	La Gomera	Barranco Majona	TB/3	Museo Arqueológico de La Gomera
GOI52	CAN.029	La Gomera	Barranco Majona	TB/001	Museo Arqueológico de La Gomera
GOI53	CAN.030	La Gomera	Barranco Majona	TB/002	Museo Arqueológico de La Gomera
HII01	-	El Hierro	La Lajura	2161 Individuo 22	Cabildo Insular de El Hierro
HII02	-	El Hierro	La Lajura	Zona 1 Corte 1 Nivel 3	Cabildo Insular de El Hierro
HII03	-	El Hierro	La Lajura	3044-4 La Lajura	Cabildo Insular de El Hierro
HII04	-	El Hierro	La Lajura	Sector 3	Cabildo Insular de El Hierro
HII05	-	El Hierro	La Lajura	Cavidad inferior A4	Cabildo Insular de El Hierro
HII06	-	El Hierro	La Lajura	Cavidad inferior Sector B2	Cabildo Insular de El Hierro
HII13	-	El Hierro	El Julán	12/8 Barrido JI-7321	Cabildo Insular de El Hierro
HII66	CAN.001	El Hierro	Punta Azul	PA-102	Cabildo Insular de El Hierro
HII67	CAN.002	El Hierro	Punta Azul	PA-125	Cabildo Insular de El Hierro
HII68	CAN.003	El Hierro	Punta Azul	PA-177	Cabildo Insular de El Hierro
HII69	CAN.004	El Hierro	Punta Azul	PA-217	Cabildo Insular de El Hierro
LAI01	-	Lanzarote	Montaña Mina	DX7	Universidad de La Laguna
LAI02	-	Lanzarote	Montaña Mina	DX202	Universidad de La Laguna
LAI03	-	Lanzarote	Montaña Mina	DX22	Universidad de La Laguna
LAI04	CAN.037	Lanzarote	Montana Mina	Nº1	Universidad de La Laguna
LAI05	-	Lanzarote	Montaña Mina	DX36	Universidad de La Laguna
LAI06	CAN.038	Lanzarote	Montana Mina	Nº3	Universidad de La Laguna
LAI07	-	Lanzarote	Montaña Mina	DX201	Universidad de La Laguna
PAI15	CAN.035	La Palma	Los Pasitos	PAS87/1/13	Museo Benehaorita de La Palma
PAI19	CAN.032	La Palma	Cueva del Agua	CA 3	Museo Benehaorita de La Palma
PAI21	CAN.036	La Palma	Salto Casimiro	SCAS71/1/054	Museo Benehaorita de La Palma
PAI32	CAN.033	La Palma	El Espigon	ES-76, VI-245, SECTOR C	Museo Benehaorita de La Palma
PAI35	CAN.034	La Palma	Huerto de los Morales	HM88/1/183	Museo Benehaorita de La Palma
TFI19	CAN.042	Tenerife	Cascajo	CA-77-23	Universidad de La Laguna
TFI20	CAN.043	Tenerife	El Capricho	CAP-152	Universidad de La Laguna
TFI21	CAN.045	Tenerife	El Portillo	POR A-71-1	Universidad de La Laguna
TFI38	CAN.039	Tenerife	Angostura	ANG-82-1	Universidad de La Laguna
TFI39	CAN.040	Tenerife	Angostura	Agostura 82	Universidad de La Laguna
TFI40	CAN.041	Tenerife	Buenavista	Buenavista 1	Universidad de La Laguna
TFI41	CAN.044	Tenerife	Cedro	CED-1	Universidad de La Laguna
TFI42	CAN.047	Tenerife	Salitre	SAL-2	Universidad de La Laguna
TFI43	CAN.048	Tenerife	Salitre	SAL-1	Universidad de La Laguna
TFI45	CAN.046	Tenerife	El Portillo	POR-R-31	Universidad de La Laguna
